# Editorial: The role of multi-modal imaging in improving refractive cataract surgery and the understanding of retinal disease

**DOI:** 10.3389/fmed.2024.1426880

**Published:** 2024-05-21

**Authors:** Xiaogang Wang, Jinhai Huang, Piotr Kanclerz, Ramin Khoramnia, Zhao Wang

**Affiliations:** ^1^Department of Cataract, Shanxi Eye Hospital Affiliated to Shanxi Medical University, Taiyuan, Shanxi, China; ^2^Eye Institute and Department of Ophthalmology, Eye and ENT Hospital, Fudan University, Shanghai, China; ^3^Key Laboratory of Myopia, Chinese Academy of Medical Sciences, Shanghai, China; ^4^Shanghai Research Center of Ophthalmology and Optometry, Shanghai, China; ^5^Hygeia Clinic, Gdańsk, Poland; ^6^Helsinki Retina Research Group, Faculty of Medicine, University of Helsinki, Helsinki, Finland; ^7^The David J. Apple International Laboratory for Ocular Pathology, Department of Ophthalmology, University of Heidelberg, Heidelberg, Germany; ^8^School of Electronic Science and Engineering, University of Electronic Science and Technology of China, Chengdu, Sichuan, China

**Keywords:** optical coherence tomography, optical coherence tomography (angiography) (OCTA), cataract surgery, retinal disease, multi-modal image

## Introduction

Cataracts and retinal diseases represent a significant proportion of ocular diseases in ophthalmology, and their incidence shows a continuous upward trend (1, 2). The advent of refractive cataract surgery and the patient's increasing demands of visual quality have led clinical practitioners to focus not only on the safety of cataract surgical procedures and the optimization of surgical plans but also to consider the specific retinal condition comprehensively. Simultaneously, they must consider whether the patients' overall health status, including systemic diseases and potential risk factors, could increase surgical risk, lead to intraoperative/postoperative complications, or worsen outcomes.

Ophthalmic multimodal imaging has bridged the gaps and deficiencies of single-modal imaging, providing crucial reference value for clinical issues in ophthalmology (3). It also aids in analyzing and assessing risk factors associated with the two major categories of diseases: cataracts and retina-related conditions.

## Overview of articles in this Research Topic

A total of seven manuscripts were received, and four were ultimately accepted for publication in this Research Topic after communication and discussion with the co-editors and undergoing a rigorous external peer-review process. The published research primarily focuses on the identification of risk factors and prognosis assessment for central retinal vein occlusion (CRVO) in young populations, intelligent analysis and evaluation of progression changes in dry age-related macular degeneration (AMD) using deep learning on optical coherence tomography (OCT) images, precise intraocular lens (IOL) power calculation using SS-OCT-based total keratometry (TK) to correct satigmatism, and evaluation of retinal vascular changes in COVID-19 patients 12 months after treatment using OCT angiography (OCTA).

CRVO occurs in 10–15% of cases in individuals under 40 years of age (4). However, there is limited reporting on this population's etiology, risk factors, and treatment prognosis. Berguig et al. analyzed data from 52 CRVO patients under the age of 40 (total 54 eyes) using multimodal imaging. They found that the main risk factors included ocular hypertension (20.4%), inflammation (20.4%), high blood pressure (14.8%), and coagulation abnormalities (11.1%). Patients with hypertension and inflammation had poorer visual outcomes. This study suggests that close follow-up and timely intervention with treatments such as intravitreal injections or retinal photocoagulation should be considered for patients with high-risk factors and poor prognosis (5).

As the life-expectancy increased and aging of the population continues, the incidence of AMD is increasing annually. Early diagnosis and treatment of AMD significantly affect the prognosis of the disease (6). However, early detection of AMD, especially non-geographic atrophy (nGA), is challenging. Hu et al. developed a two-step hierarchical neural network based on 3,401 OCT images, which showed advantages in intelligent diagnosis and evaluation of early AMD. The convolutional neural network model achieved an F1 score of 91.32% and a kappa coefficient of 96.09%. Moreover, the classification accuracy of nGA improved from 66.79 to 81.65%. This study highlights the advantage of deep learning in ophthalmic imaging recognition and classification with high efficiency (7).

Corneal regular astigmatism correction during cataract surgery is crucial in improving postoperative visual quality. Traditionally, surgeons use anterior corneal keratometry values and a fixed estimate of posterior corneal astigmatism to calculate toric IOL power; as the estimated posterior corneal astigmatism might be different from the true values, this may result in over-correction or under-correction of total corneal astigmatism in the clinic (8). Chai et al. utilized IOLMaster 700 and ANTERION swept-source OCT to calculate toric IOL powers for 56 eyes (56 patients) based on actual posterior corneal astigmatism values. They found that toric IOL calculations considering actual posterior corneal surface astigmatism data with TK could improve the accuracy of toric calculations and enhance the visual outcomes. Additionally, the calculation results based on measurements from both devices were comparable. The aforementioned study emphasizes the importance of actual posterior corneal surface astigmatism and underscores the significance of the concept of refractive cataract surgery.

Although COVID-19 primarily affects the respiratory system, some studies have utilized OCTA imaging technology to demonstrate a varying degrees of retinal vascular abnormalities in the short term (9). The long-term effects on retinal circulation require further investigation. Castellino et al. compared OCTA in of 25 patients (50 eyes) affected by COVID-19 with a healthy cohort of 28 patients (56 eyes). They found that short-term (1-month) post-COVID-19 infection eyes exhibited decreased superficial retinal blood flow and increased foveal avascular zone area, which did not improve over time (12 months). Moreover, abnormalities in retinal circulation were highly correlated with renal dysfunction, systemic inflammation, and arterial stiffness (Castellino et al.). These findings further highlight the persistence of COVID-19′s effects on systemic and ocular health, emphasizing the need for clinicians to focus on systemic and ocular conditions when managing COVID-19 patients.

## Conclusion

With the aid of multimodal imaging, the abovementioned research highlights several key points: (1) ocular diseases should not be confined solely to the eyes themselves; physicians must pay attention to their risk factors and consider the comprehensive assessment of systemic health conditions. (2) Surgeons should optimize surgical plans based on specific ocular and systemic conditions to enhance the precision and safety of the whole treatment procedure. (3) Integration of multimodal imaging with artificial intelligence can enhance the efficiency of disease diagnosis and treatment, thereby optimizing the diagnostic and therapeutic processes.

In conclusion, the comprehensive considerations mentioned above provide a solid foundation for optimizing the diagnosis and treatment of ocular diseases. We also believe that with further advancements in multimodal imaging technology, there will be deeper insights and improvements in understanding and treating significant conditions such as cataracts and retinal diseases.

## Author contributions

XW: Investigation, Methodology, Resources, Writing – original draft, Writing – review & editing. JH: Supervision, Writing – original draft, Writing – review & editing. PK: Supervision, Writing – original draft, Writing – review & editing. RK: Supervision, Writing – original draft, Writing – review & editing. ZW: Supervision, Writing – original draft, Writing – review & editing.
